# Recognising the challenges of implementing multi-centre adaptive plan of the day radiotherapy

**DOI:** 10.1016/j.tipsro.2022.01.002

**Published:** 2022-02-09

**Authors:** Amanda Webster, Helen A. McNair, Vibeke N. Hansen, Rebecca Lewis, Emma Patel, Elizabeth Miles, Emma Hall, Shaista Hafeez, Robert Huddart, RAIDER, HYBRID Trial Management Groups

**Affiliations:** a National Radiotherapy Trials Quality Assurance Group (RTTQA), University College Hospital (UCLH), London, United Kingdom; b Division of Radiotherapy and Imaging, Institute of Cancer Research, London, United Kingdom; c The Royal Marsden NHS Foundation Trust, Radiotherapy Department, London, United Kingdom; d Copenhagen University Hospital -Rigshospitalet, Department of Oncology, Copenhagen, Denmark; e Clinical Trials and Statistics Unit, The Institute of Cancer Research, London, United Kingdom; f National Radiotherapy Trials Quality Assurance Group (RTTQA), Mount Vernon Hospital, Northwood, United Kingdom

**Keywords:** IGRT, Adaptive, Bladder cancer, Plan selection, Quality assurance, Education

## Abstract

Two multicentre adaptive radiotherapy trials utilising Plan of the Day (PoD) with a library of plans were introduced in 35 centres. The common issues that arose from all centres when introducing PoD were collated retrospectively, through reviewing the data pertaining to the pre-trial and on-trial quality assurance programme. It was found that 1,295 issues arose when introducing PoD in outlining, planning, treatment delivery i.e., PoD selection, and in the overall process of delivering PoD. There was no difference in the number of issues that arose from pre-trial to on-trial. Thus, it is recommended that the implementation of PoD is supported by guidance, reviews, and continuous monitoring.

## Highlights

• The challenges for 35 radiotherapy centres introducing plan of the day were collated.
• Challenges arose in all areas of the radiotherapy pathway.
• Compliance with a protocol must be achieved so adaptive approaches achieve their aim.
• Both training and monitoring the introduction of plan of the day are recommended.

## Introduction

Radiotherapy delivery solutions continue to progress with the introduction of volumetric and advanced image-guided radiotherapy (IGRT) In 2007 the National Radiotherapy Advisory Group in the UK recommended that the future technical standard for radical treatment should be four-dimensional adaptive radiotherapy (4D-ART) [1]. Additionally, the ESTRO 2030 vision aims for “Optimal health for all together” [2] and highlights that every cancer patient should have access to state-of-the-art radiation therapy [3]. Ergo, the radiation oncology community endeavours to fine-tune treatment delivery utilising treatment images to refine Planning Target Volume (PTV) margins [4]. However, the implementation of adaptive radiotherapy remains limited [5], [6], the evidence base for patient benefit is lacking, and the focus of current research has been enabled by advanced treatment platforms which at present only a few patients have access to.

Plan of the Day (PoD) radiotherapy, where a plan of the day is chosen from a library of plans, is an accessible adaptive approach that all centres with a standard linac can utilise. However, the guidance available for centres to introduce PoD is sparse. This work aimed to assess common issues which arose when introducing two multicentre PoD trials: HYBRID (NCT01810757) was a multicentre randomised phase II study of weekly hypo-fractionated bladder radiotherapy with or without image-guided adaptive planning [7] and RAIDER (NCT02447549) was a multicentre randomised phase II trial of adaptive image-guided standard or dose-escalated tumour focused daily radical radiotherapy in bladder cancer [8]. HYBRID and RAIDER were the first international multicentre randomised trials evaluating PoD adaptive radiotherapy.

## Methods

Participating centres were issued with the trial protocol and radiotherapy guidelines before trial activation and implementation of the PoD strategy [7], [8]. Both HYBRID and RAIDER trials had pre-trial and on-trial quality assurance (QA) programmes including a process document, outlining, planning, and PoD benchmark cases. The process document detailed the procedures each centre would undertake for scanning, planning, checking, scheduling and treating the patient following the trial documentation. The pre-trial outlining and planning cases were test cases to benchmarking centres’ trial compliance. The pre-trial PoD benchmark cases included 12 test cases for centres to perform PoD selection, which was assessed against a pre-defined gold standard [9], [10]. On-trial feedback for outlining, planning and PoD selections was based on anonymised cases from patients recruited in both HYBRID and RAIDER.

### Data collection

**Fig. 1:**
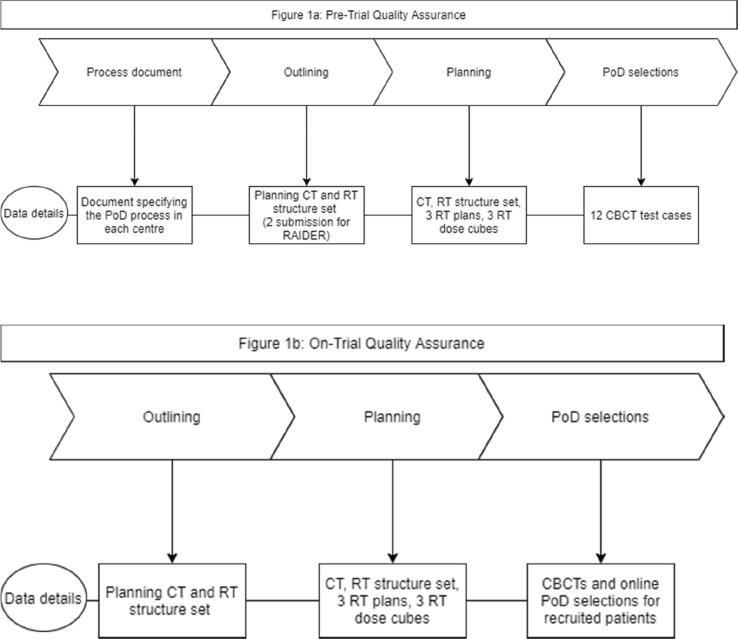
a. Pre-Trial Quality Assurance. b. On-Trial Quality Assurance.

Pre-trial and on-trial data were prospectively collected and reviewed contemporaneously to provide a reactive quality assurance programme for each trial. Retrospectively these data were collated so that the common issues which arose for centres when implementing PoD could be identified. The pre-trial and on-trial QA data included DICOM data and QA review reports, Figs. 1a and 1b. Centre variables including if they had previous PoD experience, the date of the submission of the data (for both the pre-trial and on-trial data), and the standard dose and fractionation delivered at the centre were also collected.

### Data analysis

A mixed-methods approach was used. To identify the most common PoD issues which arose in all aspects of the PoD pathway the following analysis was undertaken:

1. The total number of issues was recorded. Due to the variability of the data (e.g., DICOM data and documents), the number of issues per submission could not be calculated. Duplications were removed.
2. Issues were categorised into outlining, planning, PoD selection and PoD process groups.
3. An inductive approach was used to develop the common issues that arose during PoD implementation.
4. The practical considerations and actions recommended to centres were also recorded, from per-site QA review reports.

Descriptive statistics were used to characterise the data. A secondary analysis was undertaken to test for differences between pre-trial and on-trial issues and associations between issues and centre variables within each issue category group (i.e., outlining, planning, PoD selection and PoD process groups). Chi-square, Mann-Whitney U and Kruskal Wallis were used for the secondary analysis.

## Results

Pre-trial and on-trial data from 35 centres were reviewed. A total of 226 outline submissions, 176 planning submissions with 3 plans per submission (528 plans in total), 8,286 PoD selections from 1,267 submissions, and 43 process documents were included. Of the 1,295 issues found 160 (∼12%) issues were found in outlining, 95 (∼7%) in planning, 989 (∼76%) in PoD selections and 51 (∼4%) in the PoD process. Issues were categorised, developed and the recommendations recorded as per Table 1.

**Table 1:** Main Operative Issues when Implementing PoD.

Group	Description of Issue	Frequency
Outlining (n = 226)	Not enough bowel contoured	57
	CTV_Large does not include all of CTV_Small	47
	Not enough rectum contoured	22
	PTV_Medium > PTV_Large and/or PTV_Small > PTV_Medium	12
	No CTV_Large produced	13
	Incorrect expansions	6
	OARs contoured on incorrect dataset	3

Planning (n = 528)	Incorrect naming of plans and/or beams and/or structures	39
	D95% not growing as PTV size increases from small to medium to large	29
	OAR constraints worse for smaller plans	27

PoD selections (n = 8,286)	Plan selections not guideline compliant	119

PoD Process (n = 43)	Unable to schedule treatment	21
	Unable to schedule imaging	16
	Not considered that 2 PoD approved staff should be present for plan selection	14

The most common issues that arose for centres when introducing PoD were:

– Outlining: OARs were not outlined as per the trial protocol, e.g., bowel not being contoured superiorly enough (n = 57) and the rectum not being contoured superiorly and/or inferiorly enough (n = 22).
– Planning: Larger volumes and plans did not include excursions of smaller volumes and plans. The PTV creation for a library of plans was reliant on 2 planning CT scans. It was requested that the larger plan should encompass the smaller PTV to ensure all potential target excursions were covered. This did not occur in a total of 88 instances e.g., the large CTV did not include the small CTV (n = 47), the medium PTV was larger than the large PTV and/or the small PTV was larger than the medium PTV (n = 12), the D95% was smaller for the medium and or large plans than the smaller plans (n = 29).
– Planning: Smaller plans were less well optimised than larger plans paradoxically resulting in cases where OARs doses were higher for smaller plans compared to medium or large plans (n = 27).
– Planning: Incorrect nomenclature for plans, beams and/or structures (n = 39).
– PoD selection: Non-compliant PoD selections (n = 989) i.e., larger, or smaller plans were selected than recommended when following the PoD selection guidance.
– PoD process: Centres were unable to schedule treatment and imaging for PoD (n = 37).

In the secondary analysis, comparing pre-trial and on-trial there was no decrease in the number of issues that arose from pre-trial to on-trial. Within each issue category, there were no statistically significant associations between the centre variables and type of issue and number of issues other than in the PoD selection analysis. The PoD selection compliance decreased from pre-trial to on-trial. Higher concordance in the PoD selections in the pre-trial QA PoD training cases (88.4%) compared to online PoD selections (79.1%) (P < .001). Early identification of this precipitated iterative training including additional workshops. Results of this have been reported separately [9].

## Discussion

This work summarises 1,295 issues that arose in 35 centres introducing PoD radiotherapy as part of two multi-centre randomised trials. Even with an extensive pre-trial and on-trial QA programme, including detailed trial documentation and dedicated support [7], [8], [9], [10], there were still issues arising for all centres.

The most common issue identified was that OARs were not outlined per the trial guidelines. The most frequent deviation was an inadequate volume of bowel contoured cranially. This risks inaccurate assumption of dose to OAR, e.g., the resulting plan can underestimate the true dose to the bowel. It is not just in bladder PoD that this issue needs to be recognised but other treatment areas, including gynaecological PoD.

In 88 cases the larger PTV(s) and plans did not always include the encompass the smaller PTV in the library of plans. For clarity, this did not mean that a small plan was overall larger than a medium or large plan, but that in certain areas the smaller volumes/plans extended beyond the larger volumes/plans. In some cases, this can be due to the use of a second planning CT scan registered to the primary planning scan to outline the large PTV [8], but it can also be due to the motion and deformation which occurs in the bladder over the period required to acquire the two scans. The trial documentation included guidance, images and checklists to try and mitigate this occurring; however, it was still a frequent issue for centres. If considering introducing PoD, a process should be implemented that prevents this from occurring, e.g., using scripts that auto generates the PTVs based on the protocol, and developing a checking method at the outlining stage prohibiting the patient from progressing to the planning phase unless the outlining is confirmed compliant. This is relevant to bladder PoD and other tumour sites, including rectal PoD [11].

It was also found that smaller plans were less well optimised than larger plans in 27 cases. It should be noted that this analysis was limited to a review of the plan assessment form (PAF), and did not include a detailed dose analysis on treatment planning review software. Yet, it was found on the PAF, that the dose constraints achieved in OARs for smaller plans were worse in some instances than larger plans. This was likely due to the smaller margin plans after meeting mandatory dose constraints not being ‘pushed’ to deliver the best possible dose distribution. This was unexpected considering that in both trials the small CTV to PTV margins were 5 mm. Currently, centres are reviewing the size of their CTV to PTV margins, intending to decrease them as the utilisation of advanced IGRT techniques continues to increase [4], [12] and systems dedicated to daily plan adaptation, based on CBCT or MRI allow for smaller target volumes to potentially be treated [13]. Thus, the issue of smaller plans being less optimised is a key consideration in PoD and other advanced radiotherapy approaches, and it needs to be addressed if we are to gain the maximum benefit e.g., better clinical outcomes for patients.

In 39 cases nomenclature deviating from the protocol specified standards was utilised for plans, beams and/or structures. The trial documentation mandated consistent naming of the structures but did not mandate the naming of plans and beams. Instead, centres were required to have a process established so that the plans and beams were indicative of the size. Therapeutic Radiographers (RTTs) must select the most appropriate plan online daily. If the data is not named appropriately this can make the process cumbersome but moreover unsafe in a process that is already quite demanding on RTTs. Therefore, care should be taken to ensure that standardised nomenclature indicative of the plan size is taken when introducing PoD.

In the PoD selection process, there were 989 non-compliant selections. Non-concordant selections appeared larger than necessary in most instances. This probably reflects a ‘fear of missing’ being greater than concern regarding excessive normal tissue irradiation [14]. Using larger than necessary selections could undermine one of the tenets of this approach namely that using smaller selections can maintain target coverage with reduced toxicity due to smaller volume irradiated. Greater non-compliant PoD selections were identified in trial patients in both HYBRID and RAIDER [14], compared to the pre-accrual PoD benchmark cases. This stresses the need for iterative support to radiotherapy teams when introducing new techniques which were addressed by ensuring on-trial QA was conducted to identify issues as they arose, for which targeted support was introduced [9].

Additionally, in the PoD selection process centres struggled with the workflow e.g., scheduling imaging and treatment fields. Considering PoD was novel for most HYBRID and RAIDER centres it was recommended that centres undertook workflow test runs and/or risk assessments before treating their first PoD trial patient. Engagement with vendors was also recommended to ensure that the processes implemented were optimised considering the software and equipment available.

Of note, the purpose of both randomised trials were to compare different adaptive approaches to whole bladder radiotherapy. To minimise the effect of variation in radiotherapy planning and delivery, on the trial outcome, a rigorous QA programme was employed, as recommended [15]. Variation has been seen in oesophageal and lung trials [16], [17] and in head and neck trials failure to adhere to the radiotherapy quality assurance guideline was associated with reduced survival and local control [18], [19]. In bladder radiotherapy, this work highlights that non-compliance to the trial protocol and guidelines arose, and further work could address if non-compliant cases impact patient outcomes.

The most frequent issues arising when introducing PoD have been presented; however, within the scope of this report, it has not been possible to present the number of issues per submission due to the mixed data collection including DICOM data and process documents. Thus, this work does not address if there were trends i.e., multiple submissions with multiple issues.

## Conclusion

In introducing PoD with 35 centres there were many lessons to be learnt and it was found that challenges arise in all aspects of the PoD in the radiotherapy chain. Adaptive radiotherapy approaches are being introduced on the assumption that smaller PTV volumes will lead to less toxicity. However, the implementation of adaptive approaches needs to be safe and ensure the CTV is within the PTV. Compliance with the protocol must focus on the objective of the approach, otherwise, the trial/approach is not testing its aim, thus a QA programme is essential. This work found there was no improvement from the pre-trial to on-trial process, and the protocol compliancy for PoD selections on-trial was worse than pre-trial. Therefore, training is recommended before implementing PoD but also monitoring the implementation is essential. Most issues that arose were not complex and simple steps could be taken to mitigate them i.e., guidance, reviewing the plans, standardising nomenclature etc. This technical note should hopefully offer centres insight into the main issues which arise when introducing PoD radiotherapy so that preventative measures can be introduced in parallel.

## Declaration of Competing Interest

The authors declare the following financial interests/personal relationships which may be considered as potential competing interests:

Shaista Hafeez:

SH acknowledges support from the National Institute for Health Research (NIHR) Biomedical Research Centre at The Royal Marsden NHS Foundation Trust and the Institute of Cancer Research, London. SH reports non-financial support from Elekta (Elekta AB, Stockholm, Sweden), non- financial support from Merck Sharp & Dohme (MSD), personal fees and non-financial support from Roche outside the submitted work.

Emma Hall:

EH declares grant funding to Institution from Cancer Research UK; grant funding to Institution from Accuray Inc. and Varian Medical Systems Inc. outside the submitted work.

Other authors declare that they have no known competing financial interests or personal relationships that could have appeared to influence the work reported in this paper.
